# Safety and Efficacy of Adjunctive Therapy With Artesunate in the Treatment of Severe Malaria: A Systematic Review and Meta-Analysis

**DOI:** 10.3389/fphar.2020.596697

**Published:** 2020-11-12

**Authors:** Yuanyuan Zou, Fei Tuo, Zhiqi Zhang, Jiawen Guo, Yueming Yuan, Hongying Zhang, Zhiyong Xu, Ziyi Pan, Yexiao Tang, Changsheng Deng, Nadia Julie, Wanting Wu, Wenfeng Guo, Changqing Li, Xinan Huang, Qin Xu, Jianping Song, Qi Wang

**Affiliations:** ^1^ Artemisinin Research Center, Guangzhou University of Chinese Medicine, Guangzhou, China; ^2^ Institute of Science and Technology, Guangzhou University of Chinese Medicine, Guangzhou, China

**Keywords:** meta-analysis, severe malaria, systematic review, safety, efficacy, artesunate

## Abstract

**Objective:** The purpose of this meta-analysis of longitudinal studies is to determine the safety and efficacy of artesunate combined with other forms of adjunctive therapies for severe malaria.

**Methods:** Following the PRISMA guidelines, we searched multiple databases with the search terms “artesunate” and “adjunctive therapy” and “severe malaria” in July 2020. If the search showed a randomized controlled trial, the study was included in this meta-analysis. The random-effects model was used to calculate the combined incidence rate and relative risk or risk difference.

**Results:** This meta-analysis included nine longitudinal studies with 724 participants. We found that the mortality rates in the artesunate monotherapy group and the artesunate + adjuvant therapy group are similar (RD = −0.02, 95% confidence interval: −0.06–0.02). The incidence of adverse reactions in the artesunate monotherapy group and the artesunate + adjuvant therapy group was also similar.

**Conclusion:** No significant differences in safety and efficacy were observed between the artesunate monotherapy group and the artesunate + adjuvant therapy group. Higher quality and rigorously designed randomized controlled studies are needed to validate our findings.

## Introduction

Malaria is a life-threatening parasitic infection that is transmitted to humans through the bites of female *Anopheles* mosquitoes. Malaria is caused by five species of *Plasmodium*, namely, *P. falciparum*, *P. vivax*, *P. ovale*, *P. malariae*, and *P. knowlesi* (WHO, 2019). Out of all the malaria causing species, *P. falciparum* is responsible for the majority of severe malaria cases and deaths (Perlmann et al., 2000). The initial symptoms of malaria such as headache, fever, and chills may be difficult to detect. However, in case the patient does not receive timely and effective treatment, he may develop severe or complicated malaria, a life-threatening condition. Children with severe malaria usually have one or more of the following symptoms: respiratory distress caused by metabolic acidosis, severe anemia, and cerebral malaria. The severity of malaria varies according to the age group and the intensity of transmission in the area. In adults, severe malaria often occurs with multiple organ failure. In endemic areas, people may develop a certain level of immunity against malaria parasites, which results in future asymptomatic infections (WHO, 2019). Cerebral malaria is one of the most lethal complications of severe malaria. Varying degrees of neurocognitive impairment, coma, seizures, and other neurological abnormalities occur in cerebral malaria (Idro et al., 2006; John et al., 2008).

At present, severe malaria is treated with drugs such as chloroquine, sulfadoxine-pyrimethamine, quinine, and artemisinin and its derivatives (artemether, artesunate). However, two of the safest and cost-effective drugs, chloroquine and sulfadoxine-pyrimethamine, have lost their efficacy in most malaria-endemic countries because of drug resistance (Lin et al., 2020; Mharakurwa et al., 2020).

Adjuvant therapy includes different treatments that alleviate malaria-induced pathophysiological manifestations by directly changing the biological pathways or physiological processes associated with malaria (John et al., 2010). In the backdrop of artemisinin resistance and high mortality rate of cerebral malaria, adjuvant therapy aims to improve clinical outcomes, reduce mortality, and prevent long-term neurocognitive impairment implicated in cerebral malaria. Effective adjuvant therapy has significant benefits when used in combination with antimalarial drugs. Apart from being safe and effective, adjuvant therapy is minimally invasive, inexpensive, neuroprotective, and easy to use in clinical settings.

## Materials and Methods

### Study Search

We followed the preferred reporting items for systematic reviews and meta-analyses (PRISMA) guidelines (Higgins and Green, 2011) and used the standard methods described in the Cochrane System Review Manual to conduct a systematic review of clinical studies (Higgins et al., 2011).

### Study Selection

Studies were selected from different electronic databases, including MEDLINE, EMBASE, OVID, CNKI (China National Knowledge Infrastructure), CBM (Chinese Biomedical Database), and Cochrane Library. We selected studies that focused on the safety and efficacy of artesunate (ATS) and adjuvant combination therapy for severe malaria. The website of the World Health Organization (WHO) and clinical trial database were also checked for the unpublished or ongoing trials. The citations of selected literature were also screened to find missing studies if any. The search included only human studies that were published in German, English, or Chinese languages between January 2000 and July 2020. The medical subject heading (MESH) terms were (artesunate OR ATS) and (adjunctive therapy OR adjuvant therapy OR complementary medicine OR add-on therapy) and (severe malaria or cerebral malaria).

### Research Options

Studies included were as per the PICOS standard as described below (Moher et al., 2010).

P: Participants. Participants included in the study were severe malaria patients living in endemic countries, with no emphasis on gender, age, or type of the malaria parasite harbored. Since the definition of severe malaria was not uniform in the included studies, malaria patients who met the following four or more conditions were considered as severe malaria patients.: 1) repeated seizures (two or more systemic attacks within 24 h), 2) acute respiratory distress syndrome (ARDS), 3) impaired consciousness (Blantyre coma score <5 for children, Glasgow coma score <11 for adults), 4) repeated convulsions, 5) hypoglycemia (glycemia <40 mg/dl), 6) severe anemia or jaundice, 6) severe shock, 7) acute renal failure (blood creatinine >3 mg/dl), 8) hyperparasitemia, and 9) hyperlactatemia. Exclusion criteria included patients allergic to ATS or adjuvant therapy drugs, pregnant or lactating women, a history of chronic diseases, severe comorbidities (diabetes, heart, kidney, or liver disease), a history of receiving antimalarial medication during five days before admission, or severe malnutrition.

I: Intervention. This study only considered intravenous ATS as the main treatment drug. The brand name and dosage of the drug were not considered.

C: Comparison. Artesunate monotherapy was compared with a combination of ATS and some adjuvant therapy on the basis of safety and efficacy.

O: Outcome. Severe malaria occurs late during infection and is associated with many complications. The primary and secondary parameters in this study were efficacy and safety, respectively. The curative effect is defined as follows: 1) FCT, fever clearance time, which is defined as the body temperature drop below 37.2°C and not rising within 24 h; 2) death, severe malaria continues to worsen until death after treatment; 3) PCT, *Plasmodium* clearance time, which is defined as the parasites turning negative in the thin film of the participants after treatment. Safety is defined as the incidence of adverse events (AEs), complications, and hemodialysis rate from the beginning to the end of treatment.

S: Study. Randomized controlled trials (RCTs) evaluating the efficacy of ATS combined with adjuvant therapy in the treatment of severe malaria were included. If abstracts and meeting reports provided sufficient data on potency of artesunate and adjuvant combination therapy, these abstracts and meeting reports were also included.

### Data Extraction and Quality Assessment

Two authors independently checked the titles and abstracts generated by the electronic search. Any differences between the opinions of two authors were resolved by mutual consensus. The authors used pilot data extraction tables to collect information from each included study. The information collected includes the first author of the study, the year of publication, the age and characteristics of the participants, the study design, the characteristics of the experimental drug, the sample size of the experimental group and the ATS group, intervention measures, dosage, treatment course, outcome measurement and adverse events, confirmation of malaria, follow-up time, and reported clinical results. Among studies with overlapping study population, the study that provided the most comprehensive data was used.

The Cochrane System Deviation Risk Assessment Tool was used to evaluate the methodological quality of the included studies, which included seven parameters: 1) random sequence generation, 2) allocation concealment, 3) blindness of participants and personnel, 4) result evaluation blindness, 5) incomplete result data, 6) selective reporting, and 7) other biases (Higgins et al., 2011). The quality of each study was assessed as high risk, uncertain risk, or low risk. When we were unsure about any reported study component, the original authors were contacted by phone or email. If there was no response from the original authors, those studies were excluded.

### Statistical Analysis

Various parameters, including the risk ratio (RR) and its 95% confidence interval (CI), mean difference (MD), and standard deviation (SD) of the continuous data of each study, were recorded. If two or more studies provided the data for the same indicator, Review Manager 5.3 (Cochrane Collaboration, Oxford, United Kingdom) was used to conduct a comparative analysis directly. For dichotomous variables, the results were expressed as risk ratio (RR) and 95% confidence interval (CI); for continuous variables, the results were expressed as mean difference (MD) or standard mean deviation (SMD) and 95% confidence interval (CI). Chi-square test and I^2^ tests were used to evaluate the statistical heterogeneity between the pooled randomized controlled trials (Higgins et al., 2003). If I^2^ was between 0 and 40%, it was classified as low, and the fixed effects model was used; if I^2^ was between 40 and 100%, it was classified as high, and the random-effects model was used. The impact of the research quality on the results was evaluated using sensitivity analysis. A funnel chart was used to assess the publication bias, and a *p* value of <0.05 was considered statistically significant. Moreover, for testing the robustness of the results, we conducted a sensitivity analysis of posttreatment mortality using R Studio (R Core Team, 2015). The currently reviewed scheme was also registered with Prospero (the registration number is CRD42020197640).

## Results

### Search Results

**FIGURE 1:**
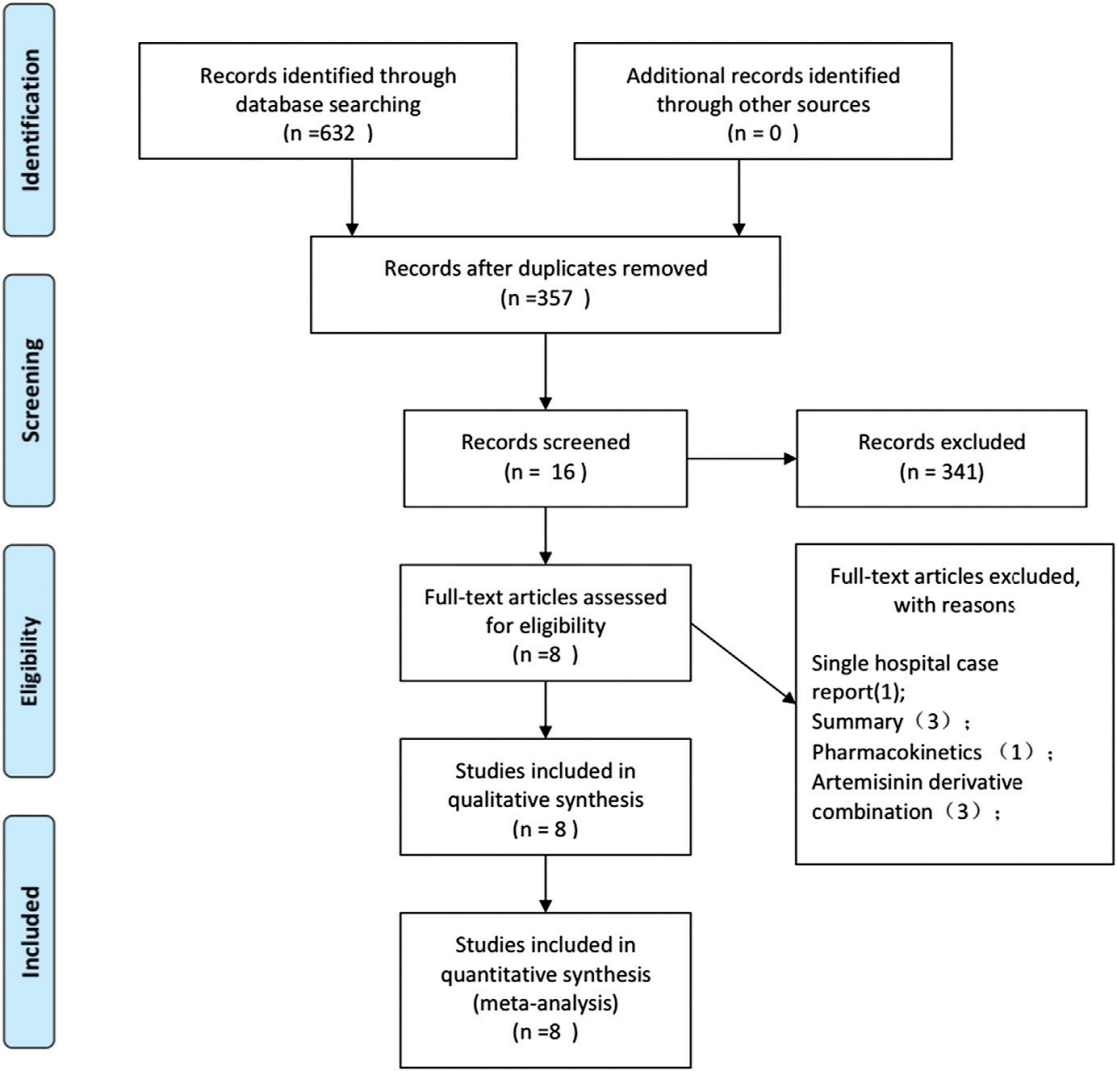
PRISMA flow chart.

The study selection process is shown in Figure 1. In the preliminary search, 632 potentially relevant records were generated. From these records, 276 studies were excluded due to duplication. In the remaining studies and abstracts, 16 were eligible for full-text reading. Subsequently, eight full-text studies were found inappropriate and hence deleted. In the end, eight eligible studies were included in this meta-analysis (Treeprasertsuk et al., 2003; Havlik et al., 2005; Charunwatthana et al., 2009; Yeo et al., 2013; Maude et al., 2014; Hawkes et al., 2015; Bangirana et al., 2018; Gao et al., 2019). The studies included one meeting abstract, supplementary information for which was found in another publication (Hawkes et al., 2015). One study was published in Chinese (Gao et al., 2019), and the rest were published in English. The eight included studies were randomized controlled trials that reported data on safety and efficacy of treatment, and three reported data on the number of people receiving hemodialysis (Treeprasertsuk et al., 2003; Charunwathana et al., 2009; Hawkes et al., 2015).

### Study Characteristics

The current review includes the results of eight randomized controlled trials. The characteristics of the included randomized controlled trials are shown in Table 1. Among the eight randomized controlled trials, two were conducted in Uganda and Thailand each, while the other studies were carried out in Congo, Papua New Guinea, and Bangladesh (two trials). In this review, we compared the findings of two studies that reported the treatment with ATS and inhaled nitric oxide (iNO) adjuvant therapy (Hawkes et al., 2015; Bangirana et al., 2018). The adjuvant therapies included in the other studies were Xiao Chai Hu (XCH) (Gao et al., 2019), levamisole (Maude et al., 2014), L-arginine (Yeo et al., 2013), N-acetylcysteine (NAC) (Treeprasertsuk et al., 2003; Charunwatthana et al., 2009), and curdlan sulfate (CRDS) (Havlik et al., 2005). All eight randomized controlled trials evaluated the safety and efficacy of the treatment administered. These studies included a total of 724 participants and were published between 2003 and 2019. The 367 cases in the control group were treated with ATS, and the 357 cases in the test group were given different adjuvant therapies based on local standard treatment. The participants or their guardians gave written informed consent for the participation in these trials, which were approved by the local ethics committees. These eight studies investigated the clinical and laboratory parameters of the participants and reported that the authors and the drug manufacturers did not have any conflict of interest. The drug manufacturer had no role in the planning and implementation of trials and protocol design.

**TABLE 1:** Characteristics of the included studies.

Study author, publication year [reference]	Country	Study population	Number (F/M)	Medication (number of patients)	Complications (number)	Deaths
Hawkes 2015	Uganda	Children	180 (78/102)	iNO+artesunate (88)	NA (5), vomiting (4), diarrhea (5), AKI (7), HG (34)	6
				Room air placebo+artesunate (92)	NA(8), vomiting(4), diarrhea(4), AKI(7), HG(29)	8
Gao 2019	Congo	Adults	87 (7/80)	XCH+artesunate (46)	NR	0
				Artesunate (41)	NR	0
Bangirana 2018	Uganda	Children	120 (50/70)	iNO+artesunate (61)	Hemoglobinuria (0), NA (5), PA (8), SA (9)	11
				Placebo+artesunate (59)	Hemoglobinuria (3), NA (8), PA (4), SA (3)	12
Maude 2014	Bangladesh	Adults	56 (16/40)	LH+artesunate (29)	NR	5
				Artesunate (27)	NR	9
Yeo 2013	Papua	Adults	8 (2/6)	Arginine+artesunate (6)	Lactic acid increased (4)	0
				Saline infusions+artesunate (2)	Lactic acid increased (1)	0
Charunwatthana 2009	Thailand+Bangladesh	Adults	108 (33/85)	NAC+artesunate (56)	ARF (14), AP (14), BWF (7), septicemia (10)	21
				Placebo+artesunate (52)	ARF (14), AP (11), BWF (7), septicemia (11)	17
Havlik 2005	Thailand	Adults	80 (20/60)	CRDS+artesunate (25)	NR	0
				Artesunate (25)	NR	0
				CRDS+artesunate (15)	ACF or/and PO (2)	2
				Artesunate (15)	ACF or/and PO (3)	3
Treeprasertsuk 2003	Thailand	Adults	85 (32/53)	NAC + artesunate (31)	vomiting (16), jaundice (13), anemia (9)	2
				Artesunate (54)	vomiting (35), jaundice (25), anemia (22)	0

XCH, Xiao Chai Hu; iNO, inhaled nitric oxide; NAC, N-acetylcysteine; CRDS, curdlan sulfate; ARF, acute renal failure; PO, pulmonary edema; AKI, acute kidney injury; LH, levamisole hydrochloride; AP, aspiration pneumonia; NA, neurologic abnormalities; PA, persistent acidosis; HG, hyperglycemia; NR, not reported.

### Trial Quality

The methodological quality of the included trials was evaluated using the Cochrane bias risk assessment tool. The findings of the methodological quality of the included trials are shown in Table 2. All the nine trials claimed to have used the random allocation method, but only six explicitly pointed out the use of the envelope double-blind method. Since blinding and other biases in the evaluation of results were not clearly described in the nine included studies, they were assessed as unclear or low risk, based on the full text. Overall, the majority of the included randomized controlled trials (87.5%, 7/8) were “low risk of bias” because they met five of the seven evaluated criteria.

**TABLE 2:** The risk of bias of the included trials.

Description of domains	Author, publication year							
	Hawkes 2015	Gao 2019	Treeprasertsuk 2003	Bangirana 2018	Maude 2014	Yeo 2013	Charunwatthana 2009	Havlik 2005
Random sequence generation	Yes	Yes	Yes	Yes	Yes	Yes	Yes	Yes
Allocation Concealment	Yes	Yes	Yes	Yes	Yes	Yes	Yes	Yes
Blinding of outcome assessment	Yes	Unclear	Unclear	Yes	Yes	Unclear	Unclear	Unclear
Blinding of participants and personnel	Yes	Yes	No	Yes	Yes	Yes	Yes	Yes
Incomplete outcome data adequately addressed	Yes	Yes	Yes	Yes	Yes	Yes	Yes	Yes
Free of selecting outcome reporting	Yes	Yes	Yes	Yes	Yes	Yes	Yes	Yes
Other sources of potential bias	Unclear	Yes	Unclear	Unclear	Unclear	Yes	Yes	Yes

Yes, low risk of bias; Unclear, uncertain risk of bias; no, high risk of bias.

### Efficacy

**FIGURE 2:**
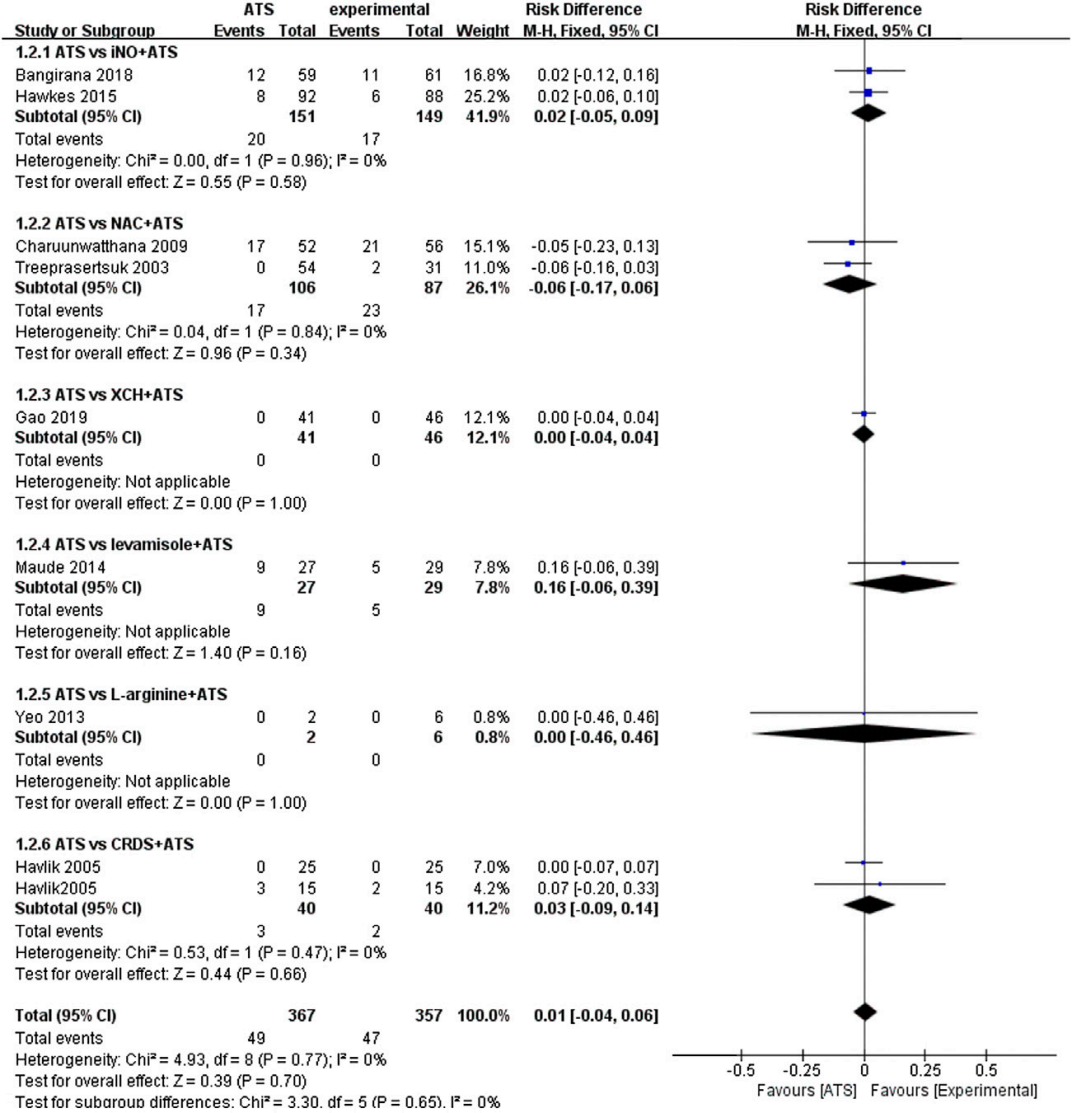
Comparison of mortality rates in ATS monotherapy group and ATS + adjuvant therapy group.

The main result of this study is the death rate of different groups. Since different adjuvant therapies were used in the included randomized controlled trials, pooled analysis is difficult to perform. We have used subgroups to compare different adjuvant therapies. There are two randomized controlled studies (n = 300) that used iNO as adjuvant therapies (Hawkes et al., 2015; Bangirana et al., 2018). We found that the case fatality rate in the ATS and iNO group (17/149) was similar to that in the ATS monotherapy group (20/151) (RD: 0.02, 95%CI: −0.05–0.09). In the study that used XCH as adjuvant therapy, we observed no significant difference in the case fatality rate between the experimental group (0/46) and the control group (0/41) (RD: 0.00, 95% CI: −0.04–0.04) (Gao et al., 2019). Similarly, we found a similar case fatality rate of L-arginine as adjuvant therapy in the experimental group (0/6) and the control group (0/2) (RD: 0.00, 95% CI: −0.46–0.46) (Yeo et al., 2013). In the two studies in which NAC was used as an adjuvant therapy to treat severe malaria, the case fatality rate difference between the experimental group (23/87) and the control group (17/106) was not significant (RD: −0.06, 95% CI: −0.17–0.06) (Treeprasertsuk et al., 2003; Charunwatthana et al., 2009). Similar death rates were also obtained with CRDS as adjuvant therapy (RD: 0.03, 95% CI: −0.09–0.14) which also appeared in the experimental group (2/40) and control group (3/40) of the experiment (Havlik et al., 2005). As per the combined analysis of all the included studies, no significant difference (RD: 0.01, 95% CI: −0.04–0.06) in the efficacy was observed between the ATS+adjuvant therapy group (47/357) and ATS only group (49/367) (Figure 2).

Fever clearance time (FCT) and *Plasmodium* clearance time (PCT) analysis.

**FIGURE 3:**
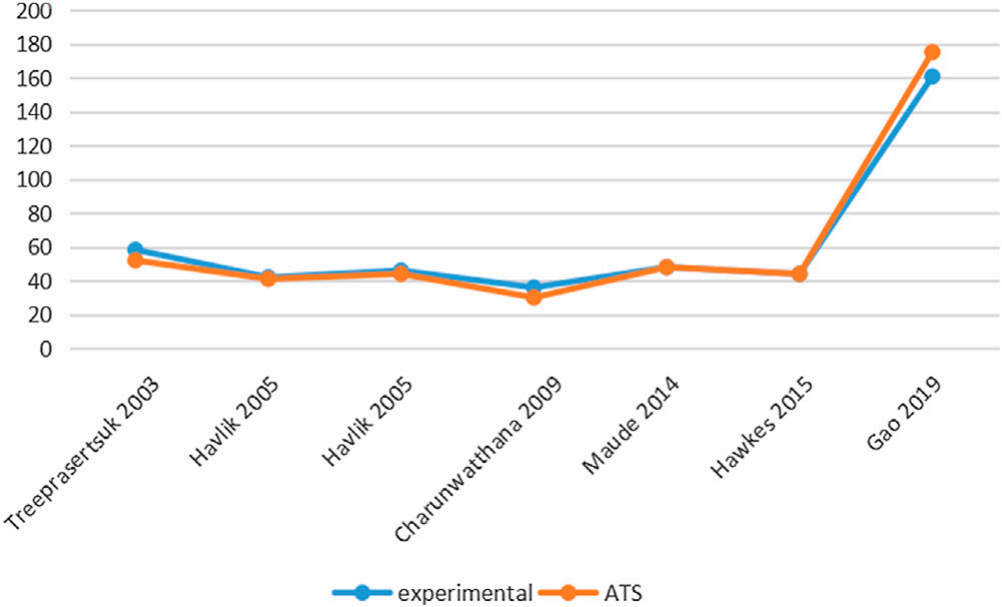
Comparison of PCT levels in ATS monotherapy group and ATS + adjuvant therapy group.

**FIGURE 4:**
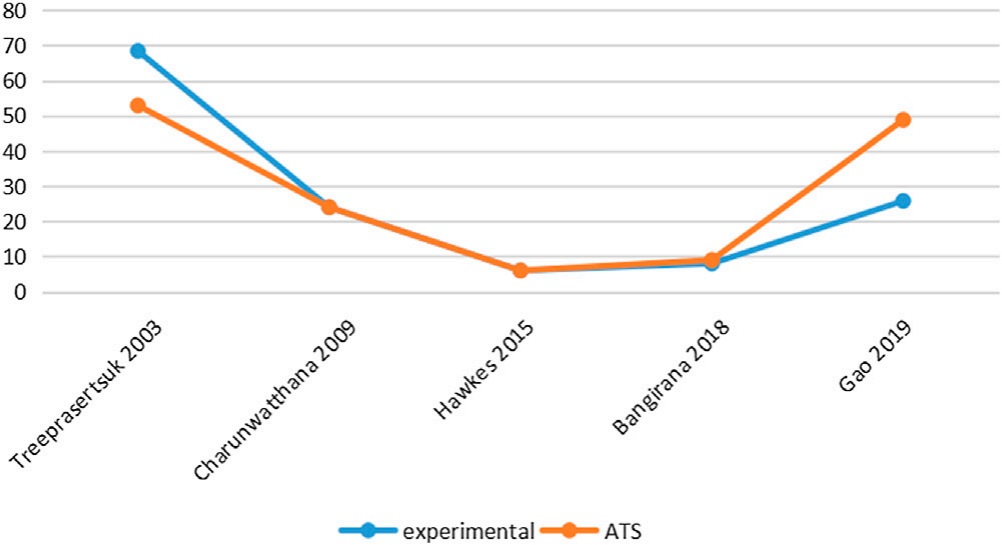
Comparison of FCT levels in ATS monotherapy group and ATS + adjuvant therapy group.

Six studies reported PCT-related data (Treeprasertsuk et al., 2003; Havlik et al., 2005; Charunwathana et al., 2009; Maude et al., 2014; Hawkes et al., 2015; Gao et al., 2019) but the difference in PCT levels among these studies was not statistically significant (Table 3; Figure 3). Similarly, five studies reported FCT-related data (Treeprasertsuk et al., 2003; Charunwathana et al., 2009; Hawkes et al., 2015; Bangiranar et al., 2018; Gao et al., 2019); however, the differences in FCT between the ATS group and the experimental group was significant only in one study (Gao et al., 2019) (Table 4; Figure 4).

**TABLE 3:** PCT levels of ATS monotherapy group and ATS+adjuvant therapy group in patients with severe malaria.

	Author, publication year					
	Havlik 2005^a^	Maude 2014^b^	Charunwatthana 2009^a^	Treeprasertsuk 2003^c^	Gao 2019^c^	Hawkes 2015^d^
Experimental group (h)	Phase IIB:46	48 (36–54)	36	58.3 ± 16.8	160.8 ± 79.2	44 (35–63)
	Phase C: 42					
ATS group (h)	Phase IIB: 44	48 (42–55)	30	52.1 ± 16.8	175.2 ± 67.2	44 (37–63)
	Phase C: 41					
*p* value	Phase IIB: NS	0.889	0.51	NS	>0.05	0.83
	Phase C: NS					

a Data are presented as median, phase IIB research subjects are patients diagnosed with severe but noncerebral malaria, and phase C research subjects are patients with cerebral malaria.

b Data are presented as median (interquartile range).

c Data are presented as median±standard deviation.

d Data are presented as median (interquartile range), NS, not significant.

**TABLE 4:** FCT levels of ATS monotherapy group and ATS+adjuvant therapy group in patients with severe malaria.

	Author, publication year				
	Charunwatthana 2009^a^	Gao 2019^b,c^	Hawkes 2015^d^	Treeprasertsuk 2003^b^	Bangirana 2018^d^
Experimental group (h)	24	25.8 ± 8.6	6(0–31)	68.4 ± 40.8	8(0–32.0)
ATS group(h)	24	48.8 ± 16.3	6(0–23)	52.9 ± 34.8	9(0–28.5)
*p* value	0.49	<0.05	0.73	NS	0.92

NS, not significant.

a Data are presented as median.

b Data are presented as median±standard deviation.

c The displayed value refers only to patients who have undergone neurocognitive testing follow-up.

d Data are presented as median (interquartile range).

### Hematology and Biochemistry Analysis

Only one study (Gao et al., 2019) did not conduct biochemical blood testing, while the other seven studies reported complete biochemical analysis. Venous blood was used to detect the hemoglobin and hematocrit, determine the plasma lactic acid concentration, and measure blood oxygen saturation. However, the differences in these parameters between the ATS group and the experimental group were not significant.

### Adverse Events

Only one of the eight included studies reported adverse events, and the incidence of adverse events difference between the experimental group and the ATS group was not significant (Hawkes et al., 2015). The complications that occurred during the treatment are listed in Table 1 **.** (The authors of each included experiment clearly stated in the original text that complications are not included in serious adverse events.)

### Research Publication Bias

**FIGURE 5:**
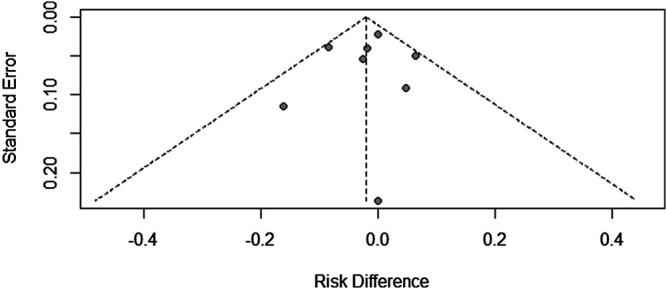
Funnel chart of included trials.

Since there were only a few included studies (<10), we created a funnel chart to evaluate the publication bias in the treatment ineffectiveness results (Figure 5). As shown in figure 5, there are four studies on the right side of the funnel chart. The heterogeneity between these studies was still not statistically significant, so the fixed effects model was used (RR = 0.8422, 95% CI: 0.5737–1.2362). At the same time, we can see that the credibility interval of the random-effects model is narrower than that of the fixed effects model, and there is no statistically significant difference. After correction, the combined effect size was still statistically significant. Therefore, the possibility of publication bias cannot be ruled out.

### Sensitivity Analyses

**FIGURE 6:**
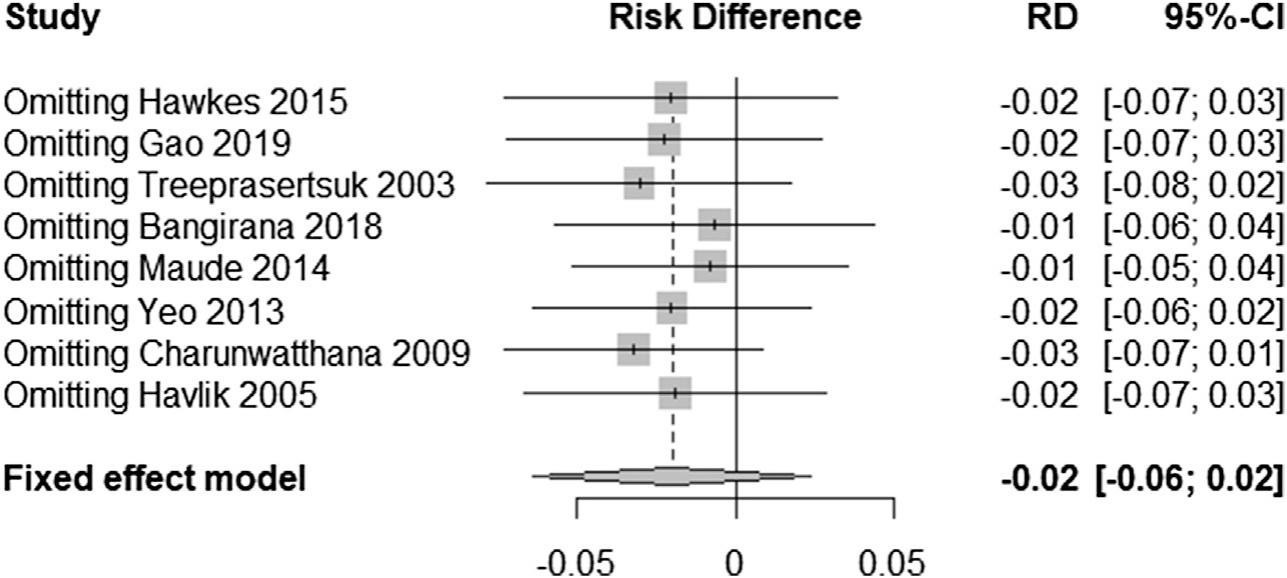
Sensitivity analysis of included trials.

In order to test the stability of the results of the included trials, we conducted a sensitivity analysis of the posttreatment mortality in the eight trials. By excluding one trial at a time and then performing a new meta-analysis of the remaining trials, we observed whether the meta-analysis results have significant changes or are stable (as shown in Figure 6). Mortality was found to be similar, with no statistically significant difference (RD = −0.02, 95% CI: −0.06–0.02).

## Discussion

Severe malaria is a complicated form of malaria mostly caused by *Plasmodium falciparum*. Severe malaria is a global public health problem that threatens the health and quality of life of the people. Although ATS is the first-line treatment of severe malaria and has high efficacy, the mortality rate in patients with severe malaria is still 8.5–18% (Wassmer et al., 2015). About half the cerebral malaria survivors may have long-term neurological sequelae and cognitive impairment (Ssenkusu et al., 2016). Severe malaria affects multiple organs in the human body and is a result of complex host-parasite interactions. Although our knowledge in understanding the pathological and physiological mechanisms of severe malaria is still limited, the onset and prognosis of severe malaria mainly depend on the complex host-parasite interactions (Wassmer et al., 2017). The pathogenesis of severe malaria is believed to be due to the combination of microvascular sequestration of infected red blood cells (iRBCs), parasite-induced immune response and endothelial activation, coagulation cascade dysregulation, and microvascular occlusion and injury; these events ultimately lead to the destruction of the endothelial barriers including the blood-brain barrier, multiple organ dysfunction, and death (Varo et al., 2018). To achieve the WHO’s goal of controlling the malaria-related morbidity and mortality as outlined in the Global Malaria Technical Strategy Report (WHO, 2015), we should use adjuvant therapies in combination with antimalarial drugs. The development of safe and effective adjuvant therapies is expected to revolutionize the treatment of severe malaria by improving the clinical survival rate and disease outcome. At present, the exact pathogenic mechanism of severe malaria is unclear, but studies have shown that immune mediators and endothelial cells play an important role in the pathogenicity and subsequent development of severe malaria. This knowledge can be exploited to develop novel intervention therapies (Glennon et al., 2018; Erice et al., 2019).

Adjuvant therapy combined with antimalarial drugs not only aims to improve the efficacy of antimalarial treatment but also reduces the incidence of malaria-related complications, including severe malaria.

Curdlan sulfate (CRD) is a sulfated 1→3-β-D glucan that can inhibit the growth of *Plasmodium falciparum in vitro* and downregulate the immune response. Sulfated glycoconjugates affect cell adhesion and rosette formation and delay schizont rupture and merozoite reinvasion (Evans et al., 1998; Kyriacou et al., 2007). The only sulfated sugar conjugate that can be used clinically is heparin. However, heparin can easily cause bleeding and is therefore not recommended for therapeutic use (WHO, 2000). The anticoagulant effect of CRD is reported to be ten times lower than that of heparin, which makes CRD more suitable as an adjuvant treatment for severe malaria as compared with heparin (Havlik et al., 2005).

Levamisole hydrochloride (LH) is a specific alkaline phosphatase inhibitor that can reduce the adhesion of *P. falciparum*-infected erythrocytes to CD36 (CD36 is one of the main vascular receptors that mediate the binding of infected red blood cells to vascular endothelial cells, which leads to isolation) by targeting extracellular alkaline phosphatase. iRBCs attach to other uninfected red blood cells to form rosettes or adhere to the endothelial cells of the capillaries. In the mouse model, it was found that LH has an inhibitory effect on the adhesion between iRBCs and endothelial cells.

The production of endothelial nitric oxide depends on the translocation of L-arginine from outside the cell to the inside through the cationic amino acid transporter-1 (CAT-1) (Yeo et al., 2008). The production of nitric oxide in endothelial cells of patients with severe malaria is reduced (Yeo et al., 2007), which can be attributed to the impaired expression of nitric oxide synthase (Anstey et al., 1996), hypoarginemia (Lopansri et al., 2003), quenching of free hemoglobin (Yeo et al., 2009), and increase in the concentration of asymmetric dimethylarginine (Yeo et al., 2010). Administration of exogenous L-arginine in adults with severe malaria improves the endothelial recovery (Yeo et al., 2007).

Inhalation of nitric oxide (iNO) in experimental cerebral malaria rodent model is reported to prolong the survival and prevent the damage to the blood-brain barrier (Serghides et al., 2011).

NAC is a well-tolerated antioxidant. It directly scavenges the free radicals and supplements glutathione and cysteine. NAC also acts on glutathione peroxidase and related enzymes and enhances peroxide clearance. In severe malaria patients, NAC may enhance the scavenging of free radicals and reduce the expression of endothelial ligands (Charunwathana et al., 2009). NAC was also found to improve erythrocyte deformation in an *in vitro* study (Nuchsongsin et al., 2007).

In general, NAC, iNO, and LH are based on antioxidants as the main starting point. Although these three methods do not show obvious advantages as adjuvant treatments, they are areas worthy of in-depth study.

Xiao Chai Hu Granules (XCH) are a kind of Chinese patent medicine, which is composed of Bupleurum, Ginger Pinellia, Scutellaria, and Codonopsis. It has the effect of relieving the surface and heat dissipation. It is often used to treat symptoms such as elevated body temperature, loss of appetite, upset, and nausea. Fever is one of the most important clinical manifestations of malaria patients. The main cause of fever is the “endogenous pyrogen” of the *Plasmodium* foreign protein reaction. The red blood cell lysate activates macrophages and T lymphocytes produced by lymphatic activating factors and the joint action of pyrogens such as tumor necrosis factor. Therefore, antipyretic in clinical treatment has become the first symptomatic treatment considered. In the past, nonsteroidal antipyretic analgesics have been used for antimalarial and antipyretic treatment, but this will further aggravate the reduction of platelets. XCH is a traditional Chinese medicine compound. Compared with traditional antimalarial drugs combined with physical cooling treatment, it shows good advantages. However, traditional Chinese medicine compounds usually have the characteristics of multiple targets. Therefore, the mechanism of XCH as antimalarial deserves more in-depth study (Gao et al., 2019.)

According to this meta-analysis, the combined therapeutic effect of different adjuvant therapies and ATS is not significantly different from the therapeutic effect of ATS alone in patients with severe malaria. This is mainly reflected from the similar number of deaths during treatment, insignificant differences in the incidence of adverse reactions, and blood biochemistry test parameters between both groups.

Although more than 50 years of clinical trials have been conducted on the adjuvant treatment of severe malaria, so far, no intervention has achieved clearly proven efficacy that it has been routinely used for severe malaria. In mouse models of severe malaria, many seemingly effective treatments have not proven effective in human trials. Although there have been no successes in the past, we have reason to be optimistic. A more comprehensive understanding of the pathogenesis of parasites and severe malaria has led to the evaluation of new therapies such as L-arginine, Chinese herbal medicines, iNO, CARDS, and NAC. These therapies have shown some promise in early trials, although there does not seem to be a great advantage for clinical endpoints other than severe morbidity or mortality. But our new research on the pathogenesis of severe malaria may advance the evaluation of new complementary interventions.

### Study Limitations

This study has several limitations. Firstly, we conducted a literature search only in German, English, and Chinese academic journals, which might have resulted in a certain degree of selection bias. Secondly, although various included studies reported no significant difference between the safety of the experimental group (ATS + adjuvant therapy) and the ATS group, the rapid development of severe malaria makes it difficult to ensure that these observations are true. Thirdly, since severe malaria affects multiple organs of the human body, measuring only the mortality, FCT, PCT, and complications does not give a completely accurate picture since changes in levels of various cytokines also occur. Lack of cytokine data in the included studies prevented us from conducting a more comprehensive meta-regression analysis. Further studies with sufficient data are needed for more in-depth analysis to explore the impact of adjuvant therapy on severe malaria and the prognostic mechanism of severe malaria. Finally, more long-term prognosis data are needed to determine the prognostic effect of ATS + adjuvant therapy in severe malaria.

## Conclusion

This study compared the safety and efficacy of ATS administered either alone or with common adjuvant therapies for the treatment of severe malaria. No significant differences were observed in the reduction of mortality, disease complications, and the incidence of adverse reactions between the ATS monotherapy group and the ATS + adjuvant therapy group. However, on the basis of the current understanding of the pathogenesis of severe malaria and the prognosis of patients with severe malaria, we conclude that common adjuvant therapies are not significantly superior to ATS. Future studies should be directed toward studying multiple targets and developing effective, minimally invasive, inexpensive, and feasible interventions to be given along with ATS for severe malaria.

## Data Availability Statement

The original contributions presented in the study are included in the article/supplementary material; further inquiries can be directed to the corresponding author.

## Author Contributions

Original idea was submitted by QW and JS. YZ carried out the review and meta-analysis with guidance from ZZ, ZP, YY, and JG. YT, HZ, ZX, WG, WW, CD, CL, XH, and QX drafted the manuscript and the remaining authors contributed with additions and amendments. All authors have read and approved the final manuscript.

## Funding

This work was supported by the Natural Science Foundation of China [Grant Number 81873218], China Postdoctoral Science Foundation [Grant Number 2019M662875], Guangzhou Provincial Science and Technology Program [Grant Number 201807010007], National “Major New Drug Innovation and Development” Science and Technology Project of Ministry of Science and Technology, People Republic of China [Grant Number 2018ZX09303008], YangFan Innovative And Entrepreneurial Research Team Project [Grant Number 2014 YT02S008], and Research Project of Guangdong Administration of Traditional Chinese Medicine [Grant Number 2019–43].

## Conflict of Interest

The authors declare that the research was conducted in the absence of any commercial or financial relationships that could be construed as a potential conflict of interest.
